# Implementing quality assurance for laboratory-based and point-of-care HIV testing in Nigeria

**DOI:** 10.4102/ajlm.v5i2.455

**Published:** 2016-10-17

**Authors:** Ado Abubakar, Samuel Peters, Oyebimpe Balogun, Sophia Osawe, Ille Mamman, Joshua Barde, Emmanuel Ojo, Nicholas Ezati, Jide Bango, Evelyn Ngige, Anthony Emeribe, Alash’le Abimiku

**Affiliations:** 1 National External Quality Assessment Laboratory, Saye Zaria, Kaduna State, Nigeria; 2 Institute of Human Virology, Maina Court, Plot 252, Herbert Macaulay Way CBD, Abuja, Nigeria; 3 Plateau State Human Virology Research Center, Plateau Hospital, Jos, Nigeria; 4 Family Health International (FHI 360), Garki Abuja, Nigeria; 5 National Tuberculosis and Leprosy Training Center, Saye, Zaria, Kaduna State, Nigeria; 6 HIV/AIDS Division, Federal Ministry of Health, Edo House Abuja, Nigeria; 7 Medical Laboratory Science Council of Nigeria, Durumi, Abuja, Nigeria; 8 University of Maryland School of Medicine, Baltimore, Maryland, United States

## HIV situation in Nigeria

**FIGURE 1:**
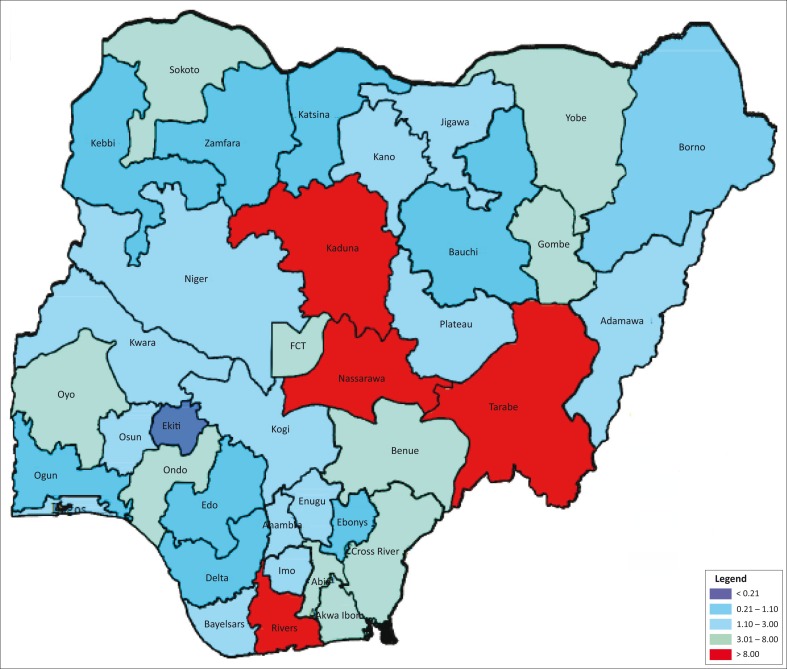
HIV prevalence in Nigeria by State.

The African National Congress sentinel survey in Nigeria has shown a steady decrease in HIV prevalence since 2001, with the following trend: 5.8% in 2001, 5.0% in 2003, 4.4% in 2005, 4.6% in 2008, 4.1% in 2010, and 3.4% in 2013.^1,2^ Despite this favourable picture, Nigeria has a mixed HIV epidemic and has populations and States where the prevalence is significantly higher than the national estimates (Figure 1). Nigeria has a population of 173.6 million.^3^ It is estimated that about 3.2 million people live with HIV in Nigeria and about 220 393 new HIV infections occurred in 2013, with 210 031 deaths from AIDS-related cases.^2^ Only about 42% of those who need antiretroviral therapy are covered. Only 4.1% of HIV-exposed infants and 17% of women who tested positive for HIV received their virological and serological test results at the appropriate time, respectively (Table 1).^2,4^ Prevention of mother-to-child transmission of HIV coverage is about 20.2%, with only 4.6 % HIV-infected pregnant women assessed for antiretroviral therapy eligibility, through either clinical staging or immune monitoring tests during the period.^2^ Viral load coverage is only 10% (Institute of Human Virology Nigeria, unpublished data), with just 419 reported laboratory-based CD4 instruments and 27 CD4 point-of-care (POC) instruments^5^ (Table1). According to the 2010 Integrated Biological and Behavioural Surveillance Survey, HIV prevalence among the most at-risk populations in Nigeria is much higher than among the general population. Prevalence is estimated at 27.4% among brothel-based female sex workers and 21.7% among non-brothel-based female sex workers. Men who have sex with men had a prevalence of 17.2% (Table 1).^2,6^

**TABLE 1:** Key characteristics of the HIV epidemic in Nigeria.

Characteristic	No. or Percentage
**HIV statistics**	
New HIV infections	~170 000 000
People living with HIV/AIDS	3 229 757
AIDS-related deaths	210 031
HIV-exposed infants who received a virological test for HIV within two months of birth	4.1%^4^
Women who were tested for HIV and received their results	17%
Pregnant, HIV-positive women who received ART to reduce the risk of mother-to-child transmission	20.2%†
Pregnant, HIV-positive women assessed for ART eligibility through clinical staging or CD4 testing	4.6%
**Diagnostic capacities**	
Reported conventional CD4 machines^9^	419
Reported POC CD4 machines^9^	27
Viral load coverage	~10%
**HIV prevalence among most-at-risk populations**	
Brothel-based female sex workers	27.4%
Non-brothel-based female sex workers	21.7%
Men who have sex with men	17.2%

## Laboratory infrastructure and HIV-related testing in Nigeria

High-quality laboratory service is essential in any HIV prevention, treatment, care, and support programme, as it ensures that clients are reliably diagnosed and treated for HIV and related opportunistic infections, such as *Cryptococcus* spp. Laboratory services are also essential for monitoring the quality of care and patient’s adherence and response to therapy. In 2015, with the help of the US Presidents Emergency Program for AIDS Relief (PEPFAR), Nigeria upgraded its laboratory infrastructure for HIV diagnosis and monitoring, including CD4, HIV serology, early infant diagnosis (EID) and viral load testing, for a total of 566 laboratories. In 2016, 428 of these laboratories, of which 76 are in tertiary and 352 in secondary facilities, are still being supported by PEPFAR. The remaining 138 primary facility-based laboratories have been transitioned to the government of Nigeria.^4^ In addition, 61 laboratories have been supported for GeneXpert^®^ (Cepheid) external quality assessment (EQA) through proficiency testing panels from the United States Centers for Disease Control and Prevention (CDC) in Atlanta. Laboratory services have been provided to over 500 000 tuberculosis patients in care and treatment.^5^ In addition, the improved National HIV/AIDS Laboratory Guideline has been distributed to all 20 universities and 47 colleges in Nigeria offering medical laboratory science programmes. Finally, close to 3000 laboratory personnel have benefitted from in-service laboratory trainings over a three-year period.

Transitioning of molecular testing in PCR-supported laboratories to the government of Nigeria is taking place to minimise the disruption of EID and viral load services, and to ensure that government of Nigeria staff are trained on and proficient in the performance of PCR-related activities. In 2016, five PCR laboratories were being transitioned to the government of Nigeria. As PEPFAR scales up viral load for patient monitoring, CD4 testing may be limited to staging and monitoring where viral load testing is not yet available. The current plan of the government of Nigeria, supported by PEPFAR, is to scale up viral load services in the 32 high burden local government areas in Nigeria and to establish a network of laboratories that can eventually support viral load testing nationally. Obviously, a robust and dependable quality assurance strategy must accompany such expansions.

## Quality assurance framework and policy for HIV laboratory and point-of-care testing

**FIGURE 2:**
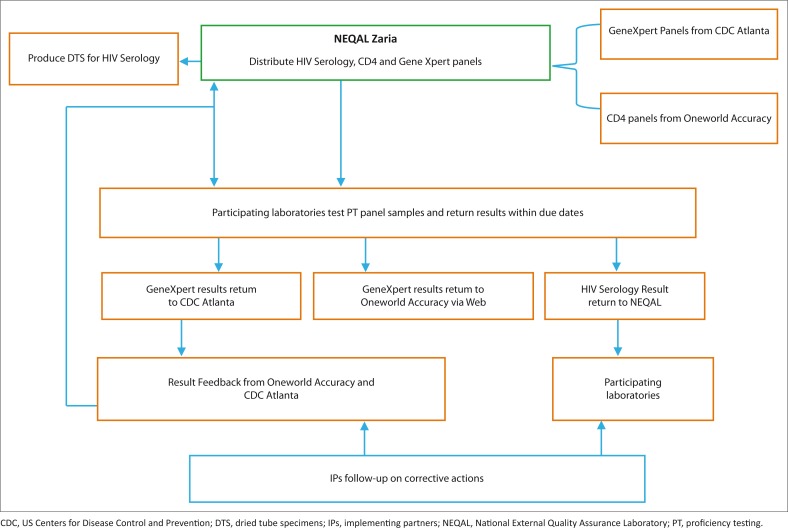
Nigeria national external quality assessment structure.

**FIGURE 3:**
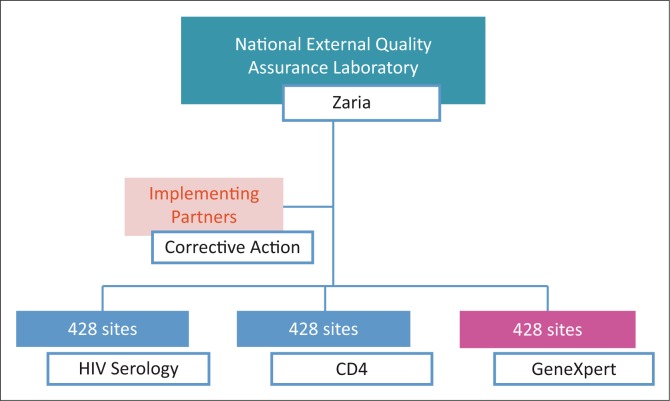
Nigeria centralised national external quality assessment model.

The national EQA framework in Nigeria is conducted through a centralised system, adopting last-mile distribution through a courier company (Figure 2). The Medical Laboratory Science Council of Nigeria, in partnership with the AXIOS Foundation, developed the national EQA centre at the National TB and Leprosy Training Center, Zaria, between 2008 and 2013, with support from the Division of Global HIV and TB of the CDC. The Institute of Human Virology, Nigeria, currently supports the Medical Laboratory Science Council of Nigeria to execute this programme. While the centre produces HIV serology panels for distribution using dried tube specimens,^7^ CD4 panels are obtained from Oneworld Accuracy (Canada). In addition, the Digital Proficiency Testing’s Oneworld Accuracy platform is used for bioinformatics and statistical analyses for both HIV serology and CD4 for the 428 laboratories still supported by PEPFAR (Figure 3). The anticipated plan is that laboratories supported by the government of Nigeria and those that are privately owned will also join the quality assurance programme to ensure high-quality practices in all laboratories supporting patient care.

## Quality assurance framework for HIV early infant diagnosis and viral load

**FIGURE 4:**
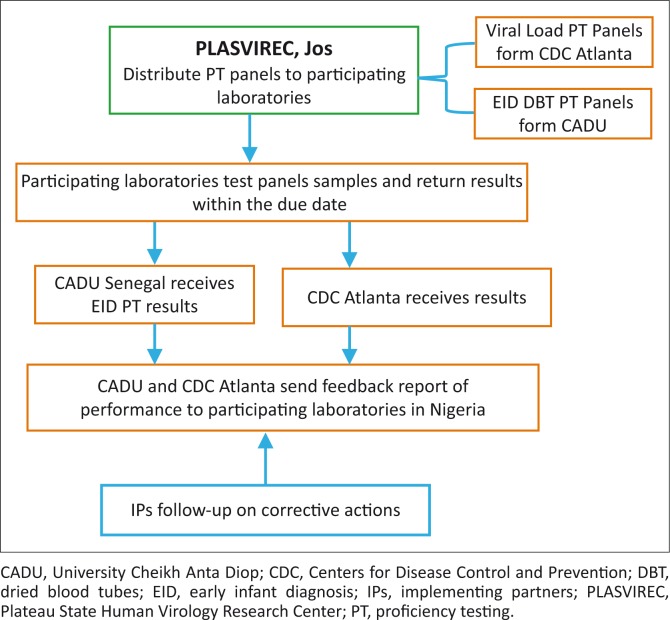
Early infant diagnosis and viral load external quality assessment structure.

CDC-Nigeria established a cost-free proficiency testing programme in Nigeria for molecular laboratory tests used for EID in 2006, and HIV viral load tests in 2010. There are currently 18 advanced laboratories participating in proficiency testing for viral load dried tube specimens and 23 laboratories participating in EID proficiency testing – a significant increase from the single laboratory, Plateau State Human Virology Research Center in Jos, where the programme was initiated in 2006. The Plateau State Human Virology Research Center now serves as a distribution centre to all participating laboratories in the country (Figure 4). The CDC has also transitioned the responsibility of the production of these proficiency testing panels to the Bacteriology and Virology laboratory at the University Cheikh Anta Diop in Dakar, Senegal, thereby building regional capacity to support countries in West Africa. The molecular laboratories in Nigeria currently participate in two proficiency testing events per year for EID and viral load. This proficiency testing is an essential component in a comprehensive laboratory quality assurance programme for the care and treatment of HIV-infected patients for any country.

## Existing quality assurance programmes

In addition to the provision of proficiency testing panels by the National External Quality Assessment Laboratory, the national quality assurance team conducts an on-site assessment by Strengthening Laboratory Management Toward Accreditation^8^-certified trainers, mentors and auditors. Rapid test kit evaluations and post-market validation of all HIV rapid test kits supplied by PEPFAR or the government of Nigeria for use in Nigeria are also an integral part of quality assurance. The implementing partners assist the State quality assurance teams in conducting HIV rapid-test-kit post-market validation in the States. In a recent drive to achieve accreditation through the World Health Organization Regional Office for Africa’s Stepwise Laboratory Quality Improvement Process Towards Accreditation^9^ and Strengthening Laboratory Management Toward Accreditation programmes, with support from the CDC, eight laboratories in Nigeria that have attained 4- to 5-star Strengthening Laboratory Management Toward Accreditation grades have been enrolled for ISO 15189 accreditation through the South African National Accreditation System. Sites that have yet to attain a 4-star grade receive continuous quality improvement activities that include training and on-site mentorship and will be presented for Stepwise Laboratory Quality Improvement Process Towards Accreditation audit after being confirmed ready by the in-country team.

## Lessons learnt

The success achieved in implementing a quality assurance programme in Nigeria can be attributed to: ownership and commitment on the part of the host institutions;^10^ technical support provided by trained Nigerian personnel in the different organisations and institutions functioning as PEPFAR and global health implementing partners; and leadership and commitment from the government of Nigeria. In our experience, an important lesson for health facilities is that quality assurance requires sustained expenditure, human and material resources, and dedication. However, it is highly rewarding for both the health institutions and their clients in the end.

The support provided by implementing partners to the government has been invaluable in developing structures, strengthening referral networks, improving antiretroviral therapy uptake, access to viral load testing, shipment, data exchange, and commodity logistics. HIV rapid-test-kit quality improvement implementation will ensure that all HIV testing points use standardised log books, run controls, participate in a proficiency testing programme and ensure that testers are certified. Provision of viral load POC testing will help to meet testing targets, as the number of PCR laboratories cannot meet the demand, especially in rural settings. It will also reduce the challenge of the logistics involved in maintaining cold chain during specimen referrals.

## Conclusion and way forward

Over a period of 10 years, the PEPFAR programme has provided the foundation in Nigeria for EQA support for CD4, HIV serology, blood chemistry, haematology, HIV viral load and EID testing to over 566 sites in Nigeria. In line with the Country Operational Plan,^11^ the government of Nigeria has already taken significant steps to support the 138 laboratories that have been transitioned to it and provide EQA support for HIV serology and CD4. The remaining 428 laboratories are still supported by PEFPAR, together with an additional 18 viral load laboratories and 23 EID laboratories, under the coordination of the government of Nigeria as part of the transition plan. Similarly, for molecular diagnostics (i.e., viral load and EID), the Plateau State Human Virology Research Center handles the enrollment of these laboratories in EQA proficiency testing from the CDC and Dakar, Senegal. The transitioning of this activity to the University Cheikh Anta Diop in Senegal will ensure reduction in costs and will foster ownership in the African sub-region.

With the current expansion of viral load testing to high HIV-burden States and local governments in Nigeria, the need for a substantial number of viral load POC testing platforms backed by a robust EQA programme in Nigeria is important for both the public and private sectors. The post-market validation of imported rapid test kits before distribution will assure that rapid test kits used in the country are of the highest quality. With PEPFAR-supported laboratories being integrated into the mainstream healthcare facility laboratories under the oversight and commitment of the federal ministry of health and Medical Laboratory Science Council of Nigeria, quality assurance uptake and ownership is on the increase, including among non-PEPFAR supported laboratories. The establishment of the national quality assurance team, which drives the post-market validation of rapid test kits, will ensure that quality assurance is a priority in both public and private healthcare facilities in Nigeria. It also sends a strong message that quality assurance standards are important to the nation and must be adhered to.

Further steps should be taken to involve State quality assurance officers in the preparation of dried tube specimens in all 36 States in Nigeria. As the country struggles to comply with the aggressive UNAIDS targets of 90-90-90, the need to increase of POC testing needs to be addressed and appropriate quality assurance put in place to ensure that the increased volume does not compromise quality. The low coverage in antiretroviral therapy, EID testing, prevention of mother-to-child transmission, CD4 POC testing, and HIV viral load testing can be improved through integrated tiered referral networks, public–private partnerships, and programme ownership by the government of Nigeria.
